# Nurse-community health mediator pairs: a promising model for promoting the health of populations in remote areas of the French Amazon

**DOI:** 10.3389/fpubh.2025.1307226

**Published:** 2025-02-25

**Authors:** Mélanie Gaillet, Margot Oberlis, Bérengère Bonot, Charlène Cochet, Estelle Jacoud, Céline Michaud, Lionel Amato, Cyril Rousseau, Cécile Caspar, Bastien Boussat, Nicolas Vignier, Loïc Epelboin, Brice Daverton

**Affiliations:** ^1^TIMC-IMAG Laboratory, UMR 5525 CNRS, Grenoble Alps University, La Tronche, France; ^2^French Red Cross, Cayenne, French Guiana, France; ^3^Prevention and Care Centres, Cayenne Centre Hospital, Cayenne, French Guiana, France; ^4^Department of Clinical Epidemiology, Grenoble Alps University Hospital, La Tronche, France; ^5^Department of Infectious and Tropical Diseases, Avicenne Hospital, Bobigny, France; ^6^Sorbonne Paris Nord University, IAME, Inserm, Bobigny, France; ^7^Department of Infectious and Tropical Diseases, Cayenne Centre hospital, Cayenne, French Guiana, France; ^8^University of French Guiana, Cayenne, French Guiana, France

**Keywords:** health mediation, health promotion, Amazonian communities, health education, French Guiana, community-health mediator

## Abstract

Multicultural Amazonian populations in remote areas of French Guiana face challenges in accessing healthcare and preventive measures. They are geographically and administratively isolated. Health mediation serves as an interface between vulnerable people and the professionals involved in their care. This approach aims to improve the health of Amazonian populations by addressing their unique challenges, including social and health vulnerabilities, as well as language and cultural barriers. A Mobile Public Health Team (MPHT) relying on health mediation was created in 2019. Comprising six nurse–community-health mediator pairs who receive ongoing specialised training, along with a coordination team of one physician and two public health nurses, the MPHT is connected to the 17 Prevention and Care Remote Centres across the territory. This article presents a community case study of the MPHT of the remote areas in French Guiana and the description of the activities of this health promotion programme in the context of the COVID-19 pandemic in 2021. The MPHT carried out health promotion initiatives, often in collaboration with partners, focusing on health priorities of the Amazonian territories. The interventions were co-designed with community leaders and local populations to ensure relevance and effectiveness. In response to the COVID-19 pandemic, the MPHT reached over 6,000 individuals in addition to more than 3,000 participants in a water, hygiene and sanitation education programme. The team performed 83 health promotion interventions on eight different topics, including 28 in the general population (922 people reached) and 55 in schools (*n* = 930). The MPHT produced 20 communication tools, which were adapted and translated into eight languages. The team also participated in managing six simultaneous epidemic events, including malaria, diphtheria, and tuberculosis. This study highlights how the combined expertise of healthcare professionals and the mediation skills of community health workers effectively addressed the specific health needs of the multicultural Amazonian populations. This model for addressing social and health inequities should encourage institutional recognition of the community health mediator model.

## Introduction

Multicultural Amazonian populations in remote areas of French Guiana face challenges in accessing healthcare and preventive measures. They are geographically and administratively isolated and have many factors of social and health vulnerability (1, 2). There are also significant language and cultural barriers, and knowledge of the healthcare system is insufficient (3–5).

As a result, the health and prevention need of the populations living in the Amazonian territories are very significant and specific.

In addition, the health care is limited, with primary care being provided by only 17 Prevention and Care Remote Centres (PCRCs). There are few health and social actors in these Amazonian territories, and they experience great instability. There is a high turnover of medical and nursing staff in the PCRCs and among the associative partners. The French healthcare system has difficulties in organising and adapting to reach these populations (1). Interventions in these areas are needed to address the obstacles encountered.

Health mediation was sought to be a valuable tool to help overcome some of these difficulties. This discipline has been deployed for nearly 20 years in French Guiana. Indeed, according to the French National Authority for Health (*Haute Autorité de Santé*), health mediation is defined as “the interface between vulnerable people who are far removed from the health system and the professionals involved in their care.” The aim of this discipline is to fight social and health inequalities and promote equal access to prevention and care. To achieve this, mediators use “their knowledge of contexts, cultures and languages to propose or negotiate solutions that are acceptable to all parties” (6, 7). WHO’s definition of community health workers make it clear that they are connectors between the primary care system and community, as health mediators in France (8). According to the different experiences reported in a WHO systematic review (8), community health workers can, depending on their country of practice, perform a variety of tasks such as contributing to the appropriate use of health services, referring patients to other services, providing health education and encouraging community members to change their behaviour, collecting and recording data, improving relations between health services and communities and providing psychosocial support. As health mediators can perform these activities, they can be compared to community health workers. In some health systems, community health workers can also provide certain clinical services, such as identifying, assessing and dispensing drugs or other pharmaceutical products, as well as providing direct care (blood tests, etc.) which is not possible in France. Clinical services can only be provided by healthcare professionals (nurses, doctors, midwives, physiotherapists and pharmacists), not by health mediators.

The support provided by health mediators should help to promote the health of the Amazonian populations by considering their specificities, i.e., social and health vulnerabilities, language and cultural barriers.

In French Guiana, this is not the first time this has been done, as health mediation has already proved its worth in various public health or research programmes and with different populations, such as projects focusing on sexual and reproductive health or malaria (9–12).

A Mobile Public Health Team (MPHT) relying on health mediation was established at the end of 2019 (13). It was composed of six nurse—community-health mediator pairs, in addition to a coordination team of one physician specialised in public health and two public health nurses with a thorough knowledge of the territory and its health issues. The missions of the team were to address the significant and specific needs of the Amazonian populations in terms of access to prevention. It aimed to respond to the prevention needs and priorities identified by the health authorities, partners, and collaborators in these territories.

The objective of this work was to provide a community case study describing the activities of this health promotion programme in the context of the COVID-19 Pandemic in 2021.

## Context

### Settings

French Guiana is a French territory in South America separated from Brazil to the South-East by the Oyapock River and from Suriname to the west by the Maroni River. It has significant geographical and cultural disparities as well as territorial administrative and economic inequalities to the detriment of the Amazonian remote populations. Indeed, almost all facilities, including the three hospitals and most social and health care organisations, are located on the coast where around 80% of the 300,000 inhabitants are concentrated (1, 2, 14).

This coastal population is multicultural, mainly made up of Guyanese and West Indian Creoles, French nationals from mainland France, people from various waves of migration (Brazilians, Surinamese, Guyanese, Haitians, Hmongs, Saint-Lucians, Chinese), and Businenge (Maroons) living in and around Saint Laurent du Maroni.

**Figure 1:**
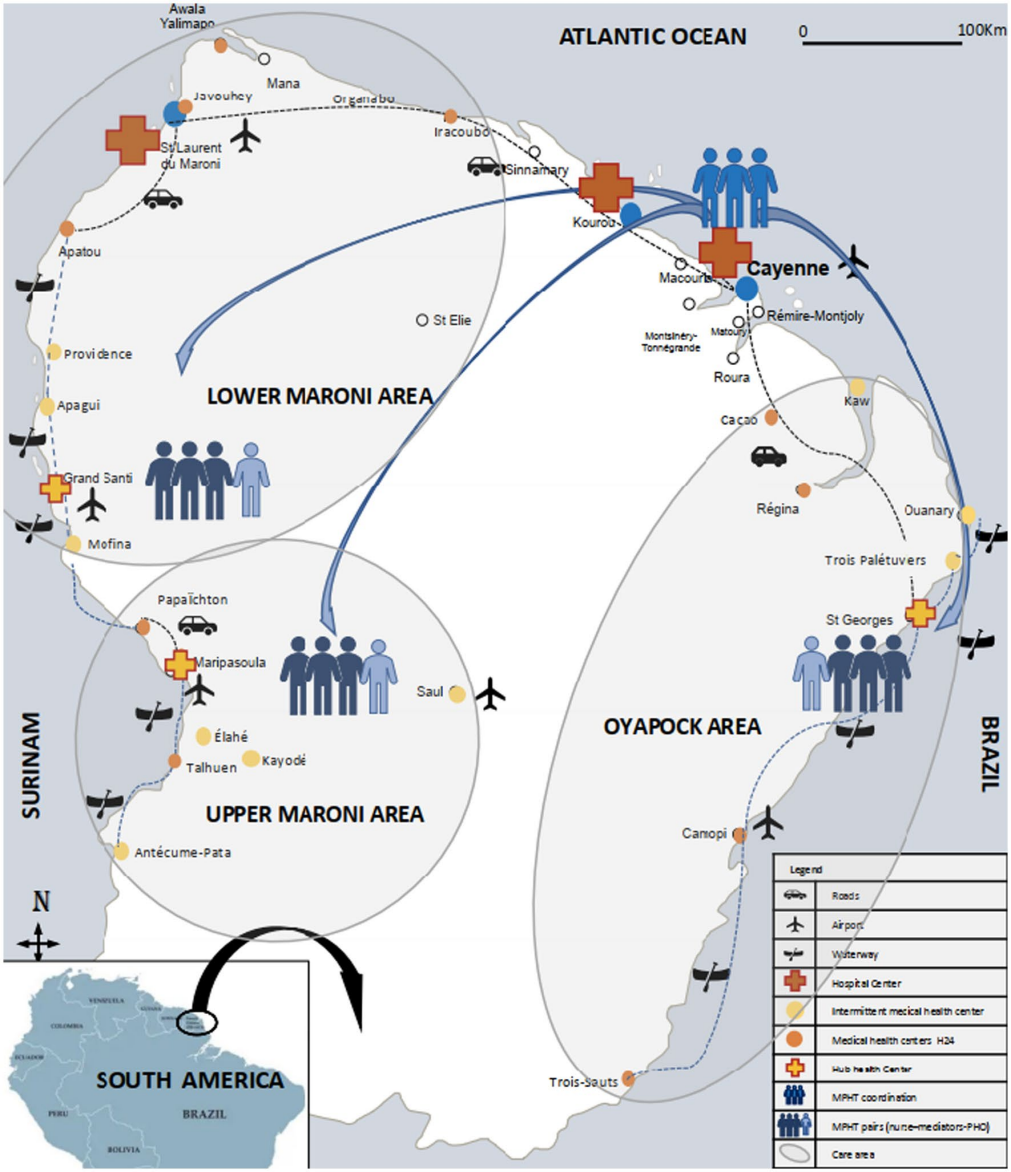
Organisation of the mobile public health team and care offer in the Amazonian territories of French Guiana, 2021.

The remote Amazonian populations are geographically isolated, with most towns and villages only accessible by pirogue along the river, or by plane. They are mainly represented by six indigenous Amerindian groups and four Businenge ethnic groups spread along the rivers, Hmongs (village of Cacao) and around 10,000 illegal gold panners, almost all Brazilian origin (3, 4) (See Appendix Figure 1; Figure 1).

### Specific needs of Amazonian populations and health services

The pathologies and social issues encountered by remote Amazonian populations include substance abuse, which is a major public health issue (although poorly documented), and a major cause of domestic violence (15). Heavy metal poisoning is also a huge public health problem (16–19). Finally, communicable infections, whether endemic or evolving through outbreaks, such as HIV infection, diarrhoea, diphtheria or malaria, also present epidemiological characteristics and management difficulties (20).

Primary health care for the Amazonian populations is provided through 17 PCRCs. The size of the PCRCs varies, ranging from a nurse-doctor-mediator trio to small hospitals with nurses, doctors, midwives, logisticians, mediators and MPHT on-site teams. Care across the remote centres is coordinated by a PCRCs coordination team composed of a multidisciplinary team based in Cayenne hospital, directed by a head trio (head physician, nurse manager, administrative manager) that also includes logisticians, medical specialists, social workers, a mediation coordinator, the MPHT coordination team and the addiction and harm reduction team. Within the PCRC coordination, a team dedicated to the fight against COVID-19 was established in 2020 and continued its mission in 2021 (21).

## Programmatic elements

The MPHT was created at the end of 2019 and was affiliated with the PRPCs. The project was funded for a renewable period of 3 years by the French Guiana Regional Health Agency (RHA, *Agence Régionale de Santé*) and the Cayenne hospital.

### Mobile public health team composition

In 2021, it consisted of a coordination team based in Cayenne hospital and three field teams located in each pivot centre (Figure 1; Appendix Figure 1).

The coordination team was made up of two public health nurses and a coordinating physician trained in public health. The latter also supervised the addiction and harm reduction team which collaborated closely with the MPHT. The coordination team recruited and trained stakeholders, defined intervention priorities based on health issues and requests from authorities, supported field teams in implementing interventions, and was responsible for promoting and evaluating the programme.

In 2019 and 2020, the field teams consisted of a nurse-mediator pair. Three additional nurses and three additional mediators were recruited in January 2021 to double the size of the MPHT field teams (See Appendix Figure 2).

### Recruitment

Mediators were recruited from the various cultural groups in the remote Amazonian territories based on their local knowledge, their integration in the community, their interpersonal skills, their motivation and their interest in prevention and health issues. Together, the six MPHT mediators spoke six local languages (2 Maroons: Ndjuka and Aluku, 3 Amerindian Wayãpi, Palikur and Wayana, and Dutch spoken in Suriname), in addition to French, Portuguese, Spanish and English.

The nurses, on the other hand, came mainly from mainland France, where they had validated their diploma as general nurses. None of them had public health training. They were recruited according to their ability to live in an isolated environment and to work in a team, their interest in working in an intercultural environment and their motivation to train in public health and health promotion.

### Training

The individuals recruited as mediators for the MPHT in 2019 and in 2021 were not previously trained in health mediation at the time of their recruitment. They participated in the “Mediation Support and Training Programme,” a three-year professional training course in health mediation deployed in French Guiana since 2019–2020 and managed by a consortium of Guianese mediation stakeholders, including the PCRCs mediation coordinator.

Training in public health, and in particular health promotion, has been provided on an ongoing basis throughout the year to all MPHT members since their recruitment. A special effort was made to ensure that they acquired knowledge on life skills based educational postures, as well as in individual and group communication techniques.

In addition, all the MPHT members received training on each of the health topics on which they were involved (See details in the Appendix).

### Organization and missions

**Figure 2:**
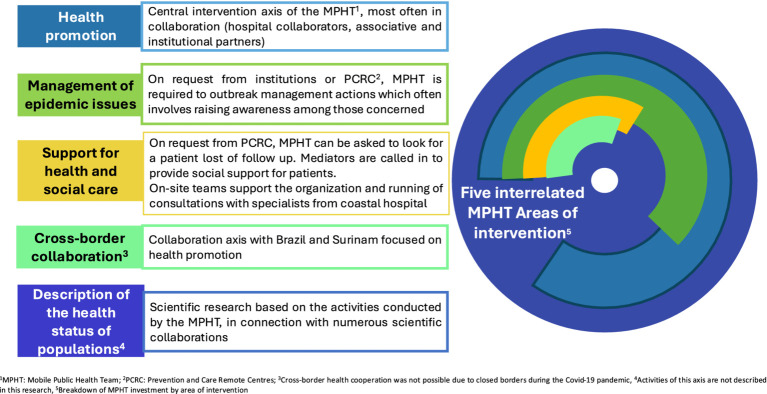
Areas of intervention for mobile public health teams. Activity interactions and variable proportion of the teams’ investment for each axis in 2021.

The MPHT’s activities were organised around five interdependent areas, as shown in Figure 2 (1-Health promotion, 2- Epidemic management, 3-Support for health and social care, 4-Cross-border collaboration, 5-Description of the populations health status).

The five MPHT intervention areas were identified at the early stage of the programme in 2019 based on needs identified by the RHA, local stakeholders and up-to-date scientific literature.

For the MPHT intervention area “health and social support,” the MPHT on-site teams worked alongside the PCRCs’ carers and mediators who operated within the PCRCs, to provide social support (i.e., support in filling administrative forms, applying for social assistance, etc.) outside the centres, thanks to their mobility in their intervention area, enabling them to reach out to patients.

The MPHT intervention areas entitled “cross-border collaboration” and “description of the populations health status “were led by the MPHT coordination team.

The “fight against COVID-19 Pandemic” and WAter, Sanitation and Hygiene (WASH) topics, were later imposed by the health context in 2020–2021 (21–23). MPHT workers collaborated with institutional and associative medico-social partners, with the Regional Education Agency (*Rectorat*) and specifically with the French Red Cross for WASH issues.

MPHT on-site and coordination teams prepared all their interventions with community leaders such as traditional chiefs, religious representatives, and key individuals involved in community life.

### Construction and monitoring of interventions

Each intervention was designed with the beneficiary populations. The teams followed a classic project methodology (24, 25). They were asked to fill a “public-health-action-form” to support the construction of any health promotion or epidemic management action.

At the end of each intervention, the teams were asked to report on it on the same public-health-action-form (number of people reached, gender, adults or children, intervention was carried out in a school setting or not, etc.). This report also included reflective feedback on the action carried out and an assessment of the impact of the action itself (satisfaction, acquisition of knowledge and/or skills).

### Data collection and analysis

To carry out this work, we collected the weekly activity reports of the MPHT on-site teams: the number and theme of activities carried out in each intervention area. The quantitative and qualitative reporting data described above were also collected except for evaluations of satisfaction measures and knowledge and skills acquisition.

The MPHT coordination team also produced regular reports on its own activities.

We analysed data on MPHT activities in 2021 encompassing all activities (See Figure 2), except the “description of the health populations status.” Qualitative data were examined using a qualitative narrative analysis of comments reported by on-site teams on action forms, while quantitative data were described in terms of percentages, means (along with standard deviations) or medians and interquartile ranges.

## Programme activities

The COVID-19 pandemic had a significant impact on the project. Firstly, it redirected some of its missions towards the fight against the pandemic in 2020 and 2021. Secondly, due to the closure of the borders with Brazil and Suriname as part of the response to the health crisis, it was not possible to invest in the “cross-border collaboration” intervention area in 2021. Thirdly, it has delayed the evaluation of its results and impact, which was due to be implemented in 2021 (21–23).

### Fighting the COVID-19 pandemic

From 2020 French Guiana experienced successive epidemic waves of COVID-19 (22). In response, the regional authorities in French Guiana (*prefecture*) launched specific measures: Borders were permanently closed, local lockdowns were imposed, domestic flights were restricted or even closed and population movements within the country or in certain regions were limited. These administrative measures had an impact on the normal course of prevention and care activities in Amazonia and have limited the MPHT’s missions.

In addition, in 2021, the MPHT has been mandated by its supervisory authorities (RHA and the PCRCs department head) to promote vaccination and “barrier gestures.” In collaboration with the PCRCs team dedicated to the fight against COVID-19, the MPHT has committed itself to the fight against the pandemic.

#### Health promotion interventions

The MPHT raised awareness about COVID-19 (identification of symptoms and what to do in case of a compatible clinical picture), promoted vaccination and emphasised compliance with social distancing measures. These activities took place at booths or during outreach activities and were accompanied by the distribution of 10,000 masks provided by the government authorities.

To support vaccination campaigns, the MPHT was tasked with conducting outreach awareness campaigns directly in the neighbourhoods where temporary vaccination activity were set up.

Actions carried out in general population are described in the quantitative approach in Table 1 (Part A).

**Table 1:** General adult population reached by health promotion actions to fight COVID-19 and made aware of water, hygiene and sanitation issues, MPHT, 2021.

A. General population adult reached through COVID-19 outreach for health promotion actions, MPHT, 2021							
Areas of care; municipality borought	Total actions; n	Out-reach awareness actions	Fixed awareness actions; n	Average of people reached per intervention n	Total of people reached; n	Population size of the municipality^2^; n	Population of the municipality reached (%)
Upper Maroni							
Maripa Soula	44	36	8	29	1,271	11,994	(10.6)
*Maripa Soula down town*	26	19	7	19	491	/	
*Amerindian territories* ^1^	18	17	1	43	781	/	
Papaïchton	10	10	0	30	300	6,212	(4.8)
Lower Maroni							
Gran Santi	56	52	4	29	1,642	8,698	(18.9)
*Gran Santi down town*	44	40	4	29	1,290	/	
*Mofina*	4	4	0	33	131	/	
*Apagui*	3	3	0	42	126	/	
*Providence*	5	5	0	19	95	/	
Apatou	2	2	0	17	34	9,381	(0.4)
Oyapock							
Saint Georges	52	51	1	46	2,374	4,188	(56.7)
*Saint Georges down town*	49	48	1	45	2,227	/	
*Trois palétuviers*	3	3	0	42	126	150^3^	(84.0)
Camopi	9	9	0	22	195	1834	(10.6)
*Camopi down town*	7	7	0	16	112	1250^3^	(9.0)
*Trois Sauts*	2	2	0	42	83	600^3^	(13.8)
Regina	3	3	0	40	121	865	(14.0)
Ouanary	1	1	0	21	21	220	(9.5)
Total	338	312	26	31	5,936	43,392	(13.7)

B. Number of general population adults reached with water, hygiene, and sanitation awareness, MPHT, 2021											
Themes of sensitization		Water transport and storage		Water treatment methods		Handwashing		Waste management		Waterborne diseases	
Areas of care; Municipality *borough*	Awareness actions mode	n^4^	(Mean)^5^	n^4^	(Mean)^5^	n^4^	(Mean)^5^	n^4^	(Mean)^5^	n^4^	(Mean)^5^
Upper Maroni area care											
Maripa Soula											
*Maripa Soula down town*	*Fixed*	89	(15)	97	(14)	0	0	8	(4)	0	(0)
	*Out-reach*	14	(5)	14	(5)	30	(8)	8	(4)	0	(0)
*Amerindian territorie* ^6^	*Fixed*	0	(0)	0	(0)	0	(0)	0	(0)	0	(0)
	*Out-reach*	58	(12)	58	(12)	46	(46)	46	(46)	0	(0)
Papaïchton	*Fixed*	0	(0)	0	(0)	6	(6)	0	(0)	0	(0)
	*Out-reach*	27	(9)	32	(8)	12	(4)	7	(4)	0	(0)
Lower Maroni area care											
Gran Santi											
*Gran Santi down town*	*Fixed*	19	(10)	19	(10)	0	(0)	0	(0)	0	(0)
	*Out-reach*	299	(15)	299	(15)	213	(21)	103	(26)	55	(28)
*Providence*	*Fixed*	0	(0)	0	(0)	0	(0)	0	(0)	0	(0)
	*Out-reach*	11	(11)	0	(0)	11	(11)	11	(11)	11	(11)
*Apagui*	*Fixed*	0	(0)	0	(0)	0	(0)	0	(0)	0	(0)
	*Out-reach*	59	(30)	32	(16)	50	(25)	0	(0)	34	(34)
*Mofina*	*Fixed*	0	(0)	0	(0)	0	(0)	0	(0)	0	(0)
	*Out-reach*	46	(46)	46	(46)	108	(54)	46	(46)	0	(0)
Apatou	*Fixed*	0	(0)	0	(0)	0	(0)	0	(0)	0	(0)
	*Out-reach*	34	(18)	34	(18)	30	(15)	30	(15)	0	(0)
Oyapock area care											
Saint Georges	*Fixed*	0	(0)	0	(0)	0	(0)	0	(0)	0	(0)
	*Out-reach*	195	(18)	195	(18)	387	(26)	0	(0)	145	(73)
Regina	*Fixed*	0	(0)	0	(0)	0	(0)	0	(0)	0	(0)
	*Out-reach*	12	(12)	12	(12)	7	(7)	0	(0)	0	(0)
Total		863	(17)	838	(16)	900	(20)	259	(20)	245	(37)

1 The Amerindian territories encompass the villages situated upstream from Maripa Soula on the Maroni River and its tributaries, which include Antecum Pata, Talhuen, Elahe and Cayode.

2 INSEE 2023 (20).

3 Data provided by the Amazon National Park of French Guiana, unpublished.

4 Number of people reached.

5 Mean of people reached per intervention.

6 The Amerindian territories include the villages located upstream from Maripa Soula on the Maroni River and its tributaries, including Antecum Pata, Talhuen, Elahe and Cayode.

The promotion of vaccination was confronted with “fake news,” i.e., disinformation messages which circulated intensively on social media in various communities in French Guiana. Most of the messages were anti-vaccination, and much of the disinformation concerned RNA vaccines. The MPHT worked with traditional chiefs and community leaders to deconstruct these false beliefs. To bolster these efforts, the MPHT created awareness-raising tools and materials adapted for different populations—adults and school-aged children—and translated them into eight languages. These included posters and flyers, audio and video messages broadcasted on social networks (26), through megaphones, on local radio, as well as games (such as the “handwashing rhyme” and others).

Vaccination was addressed in schools in Maripa Soula and Papaïchton (120 teenagers aged 13 and 14). Handwashing was discussed with children in Camopi, Regina and Gran Santi (no quantitative data available).

#### Epidemic management: identification of severe forms of COVID-19 and referral of patients requiring hospital care to a prevention and care centre

In remote areas, 12 missions were organised to assess the epidemic dynamics, inform, identify, and refer patients who needed care and were located farthest from the PCRCs in Camopi and Saint Georges on the Oyapock river, in Talhuen, Antecum Pata and the surrounding area on the Upper Maroni, and in the Lower Maroni. The MPHT on-site teams went out to meet patients by canoe and went door-to-door to reach the most isolated people. This approach was essential for reaching isolated populations.

### Description of the WASH activities

The WASH project began in September 2020 and continued in 2021 (23). It aimed to improve hygiene conditions by enhancing the management of access points to drinking water and spreading best practices, as access to drinking is a daily challenge in most communities in the remote areas. Additionally, it also sought to raise awareness and provide support to the population regarding effective practices in dealing with waterborne and vector-borne diseases, as well as the COVID-19 pandemic.

This was a regional project, carried out by the French Red Cross and funded by the RHA, the Guyanese authorities (*prefecture*), and European funds. The French Red Cross invited the MPHT to collaborate in 2021 and organised a specific training on the WASH issues for MPHT members so that they could implement the project in the Amazonian territories. Prior to commencing health promotion interventions, the teams conducted a territorial assessment on the status of access to drinking water, hygiene, and sanitation.

Building on this, the MPHT conducted 79 awareness-raising activities for the general population in 13 different locations, covering topics such as water transport and storage, water treatment methods, handwashing, waste management and prevention of waterborne diseases (Table 1, part B).

Additionally, 6 activities were carried out in schools on the same themes reaching with the support of two education games created by the teams. Eighty-two children were reached in Camopi and Saint Georges, 12 in Gran Santi, 7 in Regina.

The teams also organised one-off WASH-related activities for international thematic days such as the European Sustainable Development Week in Saint Georges, in the Amazonian territories of the Upper Maroni and in villages of the Lower Maroni, and World Water Day. Populations which, until recently, were excluded from local, national and global public health policies because of their geographical and cultural remoteness, have been included in these public health programmes.

Finally, Lower Maroni MPHT, in partnership with a scientific democracy association (La Canopée des Sciences) provided 3 days of activities and participatory workshops on the prevention of waterborne diseases to the inhabitants of numerous villages in the area. Microscopes were set up along the river. One hundred and one young adults were introduced to the use of microscopes, and the presence of microorganisms in their water.

### Health promotion actions: other themes addressed by MPHT

**Figure 3:**
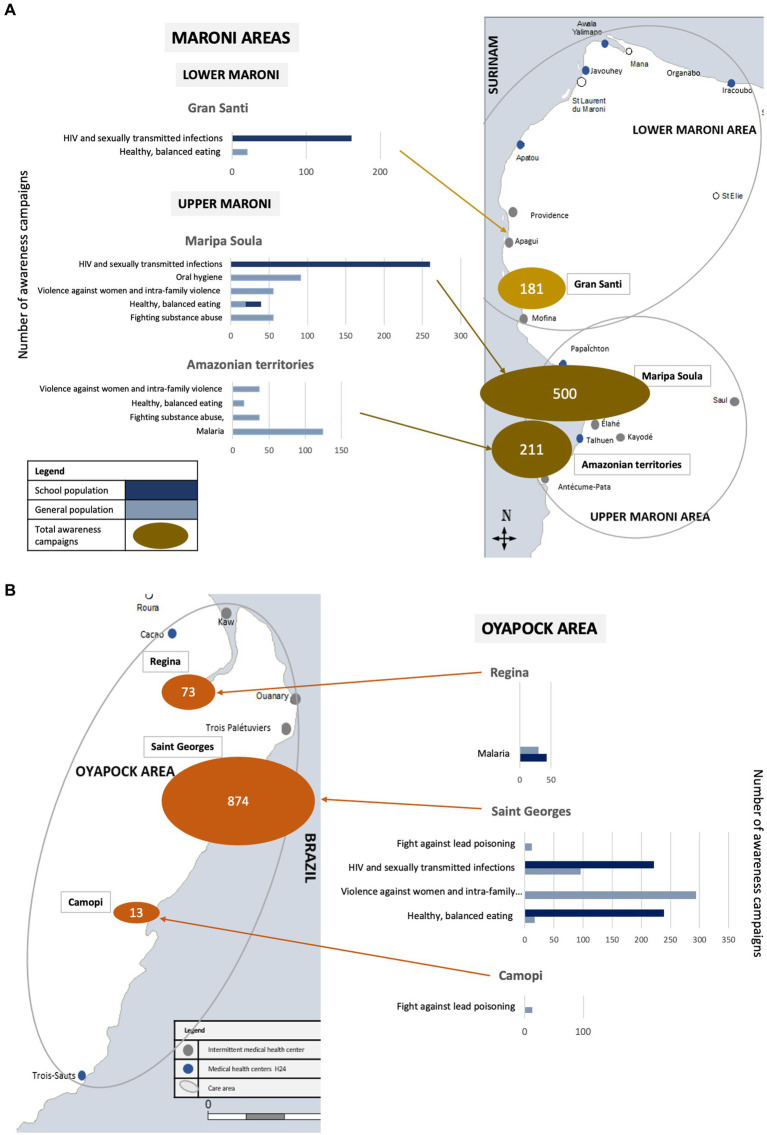
Distribution of awareness campaigns carried out by theme in French Guiana, for the general population and schoolchildren, in 2021. MPHT. **(A)** Maroni area. **(B)** Oyapock area.

Despite the pandemic, which was the focus of most efforts, the MPTH has continued to provide health promotion activities on other key issues, including the fight against drug abuse, the promotion of healthy eating, sexual and reproductive health and rights, and the fight against lead poisoning. Most health promotion projects were conducted in partnership with associations or institutions. The distribution of awareness-raising activities carried out is shown in Figure 3.

#### Fighting substance abuse, violence against women and intra-family violence issues

The traditional chiefs and the police headquarters commander of the Upper Maroni area expressed their concerns to the MPHT and the PCRCs Addiction and Harm Reduction Team, urging them to address the rise of psychoactive products consumption and suggesting awareness raising activities at community events. As a result, people were aware to harm reduction and the fight against violence against women at two events including a football tournament in Talhuen. In Saint Georges, during national day to combat violence against women and the International Day for the Elimination of Violence Against Women, almost 300 people were reached using playful health promotion tools translated and adapted to local cultures such as the “violentometer” (a visual self-assessment tool to identify the presence or threat of sexist and sexual violence in a couple’s relationship) (27).

#### Work on healthy, balanced eating and oral hygiene workshops

The issue of healthy and balanced eating in French Guiana’s Amazonian interior is particularly complex due to multiculturalism, diverse eating habits and limited access to varied diet. To address this, the MPHT organised 10 health promotion activities on balanced diet in front of Maripa Soula health centre, in Talhuen, in Gran Santi and in Saint Georges, collaborating with the diabetes and nutrition team of the Cayenne hospital. They used an adapted message to local eating habits and the available products. To our knowledge, this was the first awareness-raising campaign on this complex issue. It was enthusiastically received by the beneficiaries.

During the national gastronomy week, the teams conducted also school awareness activities, one on healthy balanced eating and four on oral hygiene (almost 300 children were reached in Maripa Soula and Saint Georges).

The teams also organised five awareness-raising activity on oral hygiene using playful health-promoting tools like toothbrushes and giant teeth on the Upper Maroni and Oyapock (more than 100 people were reached).

#### Promotion of screening for sexually transmitted infections, and education on emotional and affective life in schools

Sexually transmitted infection prevention campaigns have been run for 5 to 10 years in Saint Georges and Maripa Soula. These campaigns are supported by local associations working on these issues. The MPHT has stepped up these initiatives. In these two cities, MPHT teams participated in World AIDS Day raising awareness and screening for HIV with Rapid Diagnostic Test in collaboration with local partners. The festive nature of the event attracted many villagers and helped overcome cultural taboos.

In schools, the Oyapock MPHT conducted 7 interventions, reaching 220 pupils in Camopi, Saint Georges and Regina on puberty, managing emotions, and respecting intimacy. The Upper Maroni team led 34 interventions, reaching 260 adolescents on sexually transmitted infections and early pregnancy, while the Lower Maroni team held 6 interventions in the Gran Santi middle school (more than 160 adolescents were reached). Three new tools were developed to support these interventions.

Lastly, the Maroni MPTHs joined the Ministry of Health’s “Tumeplay program” (28), which focused on reducing new HIV infections, sexually transmitted diseases, and unwanted teenage pregnancies.

#### Fight against lead poisoning

The RHA has embarked on an ambitious project to combat lead poisoning. Food seems to be the main source of poisoning, with meat contaminated by lead shot and manioc containing significant concentrations of lead. Amazonian populations are particularly affected due to their lifestyle. The MPHT was asked to develop pilot project in the municipality of Camopi. An initial awareness-raising mission was conducted at the end of 2021 to evaluate detection and awareness tools.

#### Malaria clusters

The team developed a malaria awareness activity that was regularly presented in the waiting room of the PCRCs of Talhuen and Maripa Soula.

Additionally, the Oyapock team educated 30 pupils about the fight against malaria in Regina. The malaria educational game called “the goose game” *, developed by the Saint Georges team, was used as a teaching tool in schools during themed health promotion interventions.

*This is a classic “game of goose” in which each square has been modified to be illustrated by a malaria-related image (a mosquito, a parasite, an illustration of a symptom, a mosquito net, etc.). Each square is assigned a question linked to the illustration it bears. If the person on the square answers the question correctly, he or she can play again.

### Management of epidemic issues

**Figure 4:**
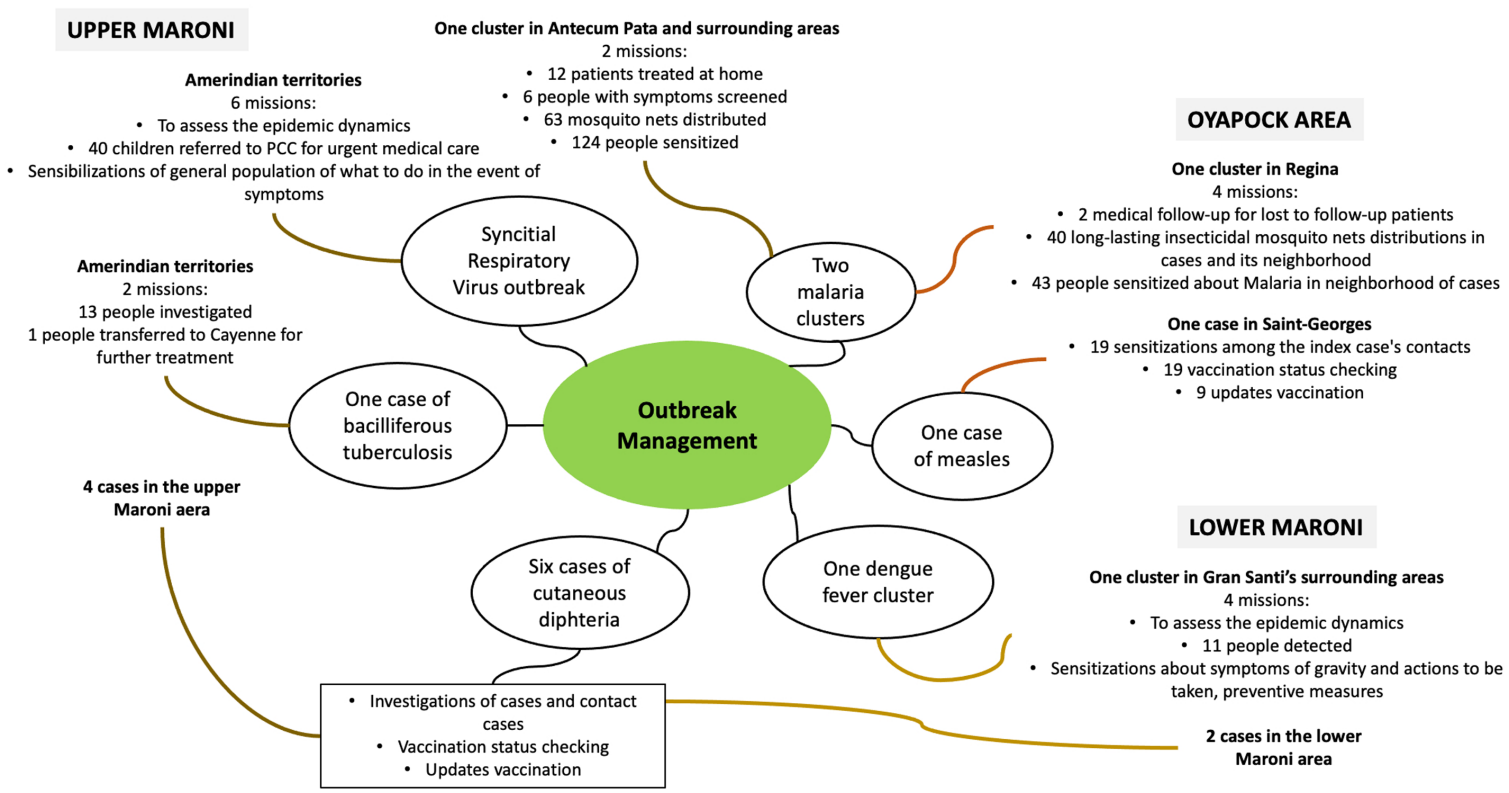
Description of MPHT management of epidemics in 2021.

After systematically working with community leaders of the affected populations to prepare interventions, with the support of mediators, the MPHT went into the neighbourhoods and patients’ homes to deliver specific care and raise community awareness (Figure 4; Appendix).

### Support for health and social care in prevention and care remote centres

Field teams ensured adherence to schedules and protocols for specialist consultations reminding patients of appointments (via telephone or home visit). MPTH supported four diabetes and four urology consultation missions in Maripa Soula and Saint Georges, with follow-up visits for 50 patients in collaboration with the diabetology team. Mediators accompanied patients during consultations and provided *ad hoc* support for infectious diseases consultations.

Additionally, the mediators provided 232 social supports reaching patients outside the PCRCs in Saint Georges, Maripa Soula and Gran Santi.

## Discussion

In 2021, the MPHT public health initiatives in French Guiana, in the Amazon territories, have demonstrated considerable value in reaching and engaging diverse multicultural populations that are distant from healthcare and prevention services. The project’s success can be attributed to several key factors, including its rapid recognition by local communities and partners, the strategic composition of the teams and its mobility and adaptability.

The combination of medical professionals and community-based mediators is a highly effective model for reaching isolated or underserved populations. Health interventions in multicultural and remote areas benefit significantly from the inclusion of individuals who are embedded within those communities and who possess intimate knowledge of local customs, languages, and challenges. This model also emphasises the importance of training health mediators and providing them with ongoing professional development to ensure they are well-equipped to handle the complexities of their roles. The MPHT’s mobility also proved to be a significant strength. The ability of teams to travel to isolated communities and conduct door-to-door visits ensured that health services reached populations that otherwise might not have had access.

### The recognition of the MPHTs value by populations, and associative and institutional partners

The recognition of the MPHT project’s value by local populations and institutional partners reflects their strong interest in and satisfaction with its initiatives. Feedback from communities and stakeholders underscores the project’s significance, as evidenced by the increasing demand for collaboration. For instance, the French Red Cross invitation to collaborate on the ambitious WASH project and the Regional Health Agency’s request to lead a new initiative on combating lead poisoning (29) demonstrate the high level of trust placed in the project. Furthermore, the traditional chiefs of Talhuen asking the Upper Maroni team to address the sensitive issue of alcohol abuse highlights the considerable trust indigenous populations place in the MPHT (30); Alcohol consumption, which is closely tied to cultural practices and social issues such as violence and suicide, remains a challenging topic within Amerindian societies (31).

### The strength of the MPHT lied in its composition of nurses and mediators from local communities

A distinctive feature of this project is its innovated model which pairs nurses with mediators from local communities. In France, mediators are not trained or authorised to provide clinical care, unlike in other countries where community health workers can have this skill (8). It was therefore essential for the mediators to work with healthcare professionals to provide out-of-home care in French Guiana. In addition, research suggests that integrating community health workers into healthcare teams can significantly improve health outcomes. Brazil has been experimenting for several years, with health promotion organisation models involving nurses, nurse auxiliaries and doctors, as well as community health workers. Evaluation of these schemes has shown that integrating community health workers into primary healthcare teams can improve the overall health of populations (32) and is a factor in the effectiveness of their interventions (33). In our model, nurses contribute their medical and technical knowledge to develop health promotion approaches and manage epidemics. The mediators’ knowledge of the area, communities, religious leaders, customs and social structures has enabled the teams to adapt their interventions to the specific needs of the populations, while respecting their beliefs and cultures (7, 34). Their knowledge and understanding of local contexts helped build trust and facilitated the care relationship, which is crucial for ethnocultural understanding and delivering high-quality care and preventive action (35, 36). Moreover, languages spoken by the communities are closely interwoven with beliefs, behaviours and convictions. The mediators’ fluency in local languages played a crucial role in overcoming the cultural and linguistic barriers that contribute to misunderstanding between populations and the health centre caregivers, including MPHT nurses (36–38).

### Mediators professionalisation and training

The high level of competence demonstrated by the mediators is a direct consequence of their professional training and ongoing support. Since 2019, they have benefitted from training programmes based on the missions clarified for mediation by the Word Health Organisation and the French Health Authority (6, 7). The quality of training and continuous support development has enhanced their skills and confidence, positioning them as effective bridges between healthcare providers and the communities they serve (7, 33, 39, 40).

### The team’s mobility enabled nurses and mediators to carry out rapid “out-reach” actions and overcome the geographical barriers that isolate populations from care and prevention

Another major factor in MPHT’s effectiveness is its mobility, allowing teams to reach out to isolated populations and thus overcome geographical barriers. This enables MPHT to travel to isolated villages along rivers, go door-to-door. The size of the on-site teams, with four agents detached from routine care, also enables them to respond quickly to urgent needs. The team can split in two, with one team with ongoing activities, the while the other focuses on urgent needs. The MPHT is therefore an interesting interventional model in the fight against malaria, especially at the time when French Guiana is in the elimination phase. This implies the need for adapted and reactive responses malaria cases (41).

### MPHT proposed an interesting model for combating the COVID-19 pandemic

The Covid-19 Pandemic posed substantial challenges to the MPHT’s operations, but the project demonstrated remarkable adaptability. The teams worked closely with community leaders to provide health information, to respond to fears and questions, and to address misinformation that was widely circulated among the population.

### Constraints and limitations

Despite the MPHT’s success, several constraints impacted its effectiveness. One significant limitation was the impact of the COVID-19 pandemic. The lockdown and restrictions on movement of people, together with the repeated closure of schools, limited the number of interventions in schools and in remote areas. Many specialist missions were cancelled, and cross-border cooperation came to a complete standstill. Finally, the turnover of MPHT nurses in 2021 was high, in the context of the traumatic confinements, resulting in “isolation upon isolation” for the teams deployed in the remote territories. The MPHT coordination team has adapted to meet the intense recruitment, training, and support needs in this context. Permanent mediators, whose roles were continuous, participated to ensure the continuity of the MPHT missions during this difficult period.

Additionally, the retrospective nature of this study posed a challenge in collecting comprehensive data. Some information was missing. Some of the activity reports submitted by the teams were insufficiently complete. The context of the COVID-19 pandemic and the urgency of certain situations, as well as the impact of the constraints on the teams’ missions described above, also affected the quality of some activity reports, and hence on this analysis. However, the monitoring tools implemented by the MPHT coordination team have reduced this difficulty.

Furthermore, this is a description of the project’s activity. It was not possible to measure its impact. In fact, although each intervention should have been accompanied by an evaluation (satisfaction, acquisition of knowledge and/or skills), it was not possible to evaluate these data, as they were insufficiently structured and sometimes missing. In the future, it will be important to complement this initial analysis.

## Conclusion

The MPHT is an innovative system that pairs nurses with community mediators to address the public health needs of isolated multicultural populations in French Guiana. It provides a compelling model for tackling current and future global challenges related to ecological changes and population movements, such as malaria eradication and pandemic response.

## Data Availability

The original contributions presented in the study are included in the article/Supplementary material, further inquiries can be directed to the corresponding author.
