# Supplementary figures and images for: Nurse-community health mediator pairs: a promising model for promoting the health of populations in remote areas of the French Amazon
Source: Front Public Health. 2025 Feb 25;13:1307226. doi: 10.3389/fpubh.2025.1307226 (PMC11894573; doi:10.3389/fpubh.2025.1307226)

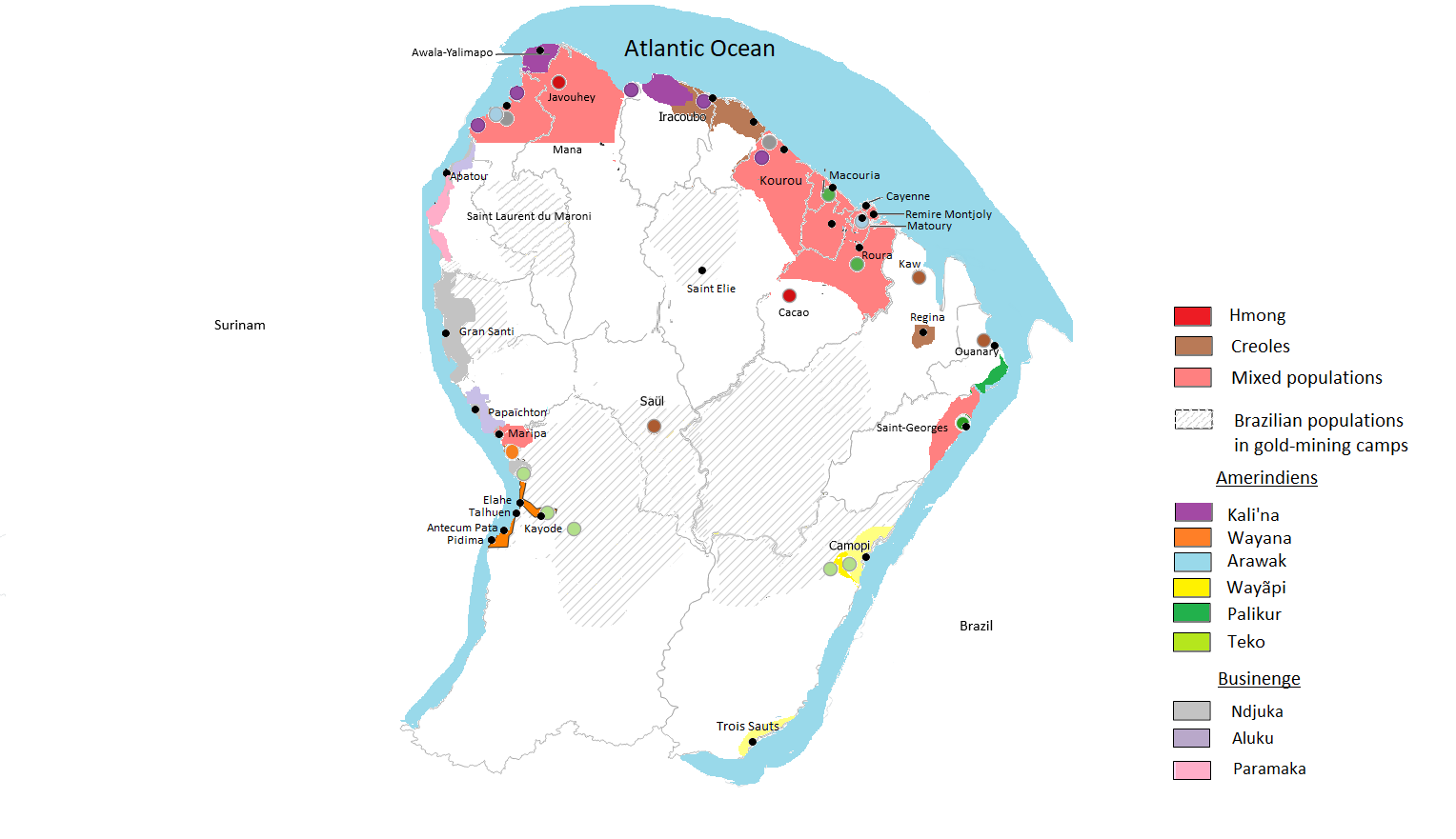

Supplement: Supplementary file 1 [file Image_1.png]

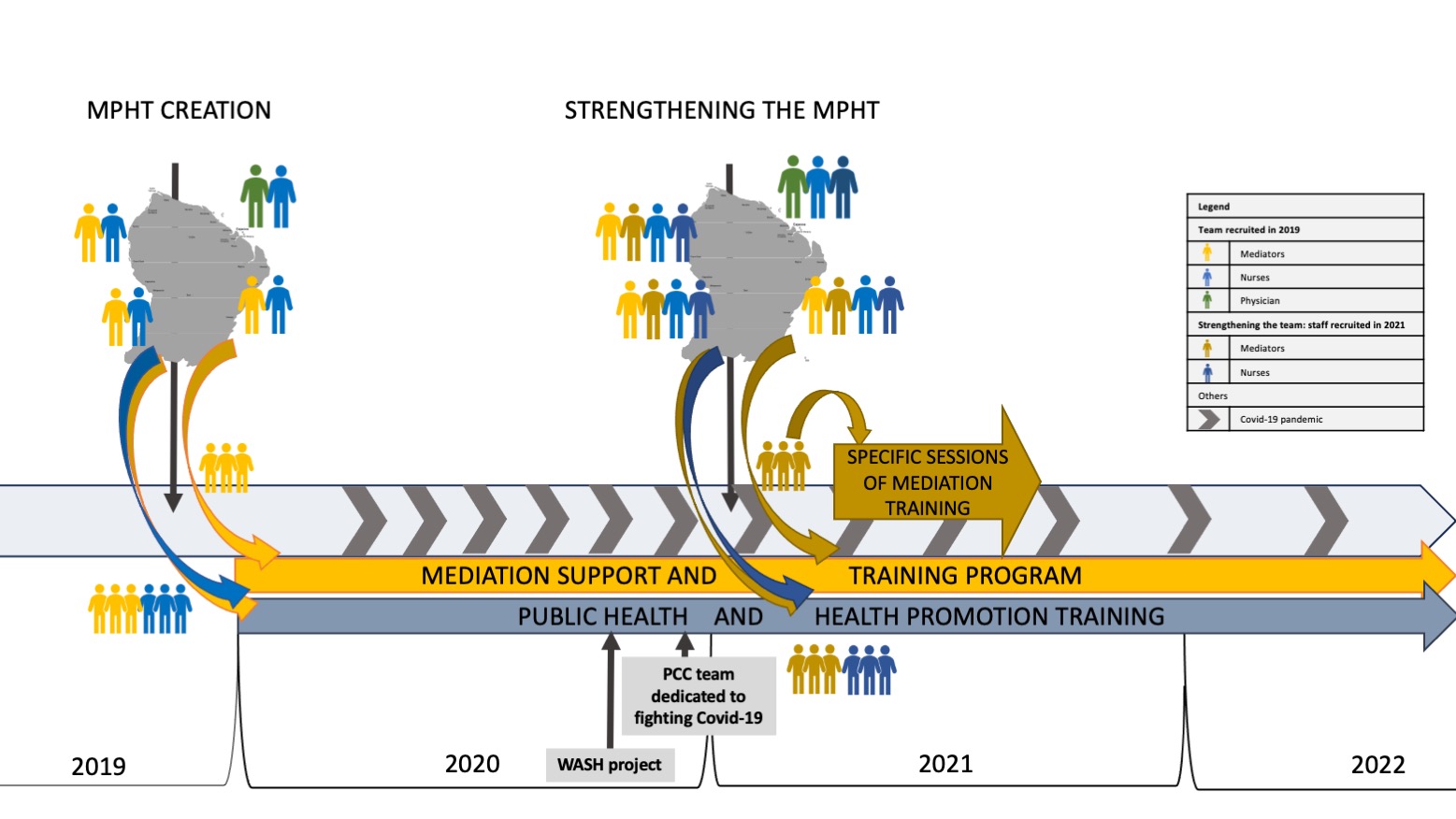

Supplement: Supplementary file 2 [file Image_2.jpeg]
