# Supplementary material for: Nurse-community health mediator pairs: a promising model for promoting the health of populations in remote areas of the French Amazon
Source: Front Public Health. 2025 Feb 25;13:1307226. doi: 10.3389/fpubh.2025.1307226 (PMC11894573; doi:10.3389/fpubh.2025.1307226)
Supplement: Supplementary file 3 [file Table_1.docx]

**Supplementary appendix**

**Appendix 1. Public health and health promotion training for the three MPHT on-site teams**

Training in public health and health promotion has been offered on an ongoing basis throughout the year to all MPHT members since their recruitment. A special effort was made to ensure that they acquired knowledge and skills in educational postures based on psychosocial skills, as well as in individual and group communication techniques. These courses were provided by the Regional Health Education and Promotion Agency (*Instances Régionales d'Education et de Promotion de la Santé*).

In addition, all MPHT members received training in each of the health topics on which they were involved. These courses were provided by partners and collaborators specialized in their area.

They were trained in:

- Fight against drug abuse (by the addictology and harm reduction team of the PCCs)

- Fight against violence against intra-family violence (by a partner association)

- Healthy, balanced nutrition and oral hygiene (by a diabetologist from Cayenne hospital)

- Fight against sexually transmitted infections, and emotional and affective life education in schools (by a partner institution)

- Fight against lead poisoning (by the Regional Health Education and Promotion Agency)

- Fight against malaria and diphtheria (by the PCCs infectiologist)

In addition, in 2021, the MPHT has been mandated by its supervisory authorities to promote vaccination and "barrier gestures". Teams were trained on this topic by PCCs team dedicated to the fight against Covid-19.

The Red Cross organized specific training on the WASH issues for MPHT members. In addition, interventions were prepared and implemented in close collaboration.

**Appendix 2. Management of epidemic issues**

After systematically working with community leaders of the affected populations to prepare interventions, with the support of mediators, the MPHT went into the neighborhoods and patients' homes to deliver specific care and raise awareness among the population

***Six cases of Corynebacterium diphtheriae***

Six cases of *Corynebacterium diphtheriae* diagnosed in a PCC were investigated by the MPHT on-site teams. They also screened and updated the vaccinations of patients and their families while on patrol. The teams are now well used to this exercise, which is carried out in collaboration with the PCCs caregivers, the PCCs infectiologist, the Cayenne hospital laboratory, and the National Reference Center for Corynebacteria of the *diphtheriae complex*. This involved six skin forms, half of which carried the tox gene.

***Investigation around a case of bacilliferous tuberculosis***

One case of bacilliferous tuberculosis was diagnosed in a patient living on the Upper Maroni area, close to the village of Antecum Pata. An investigation around the case was organized by the MPHT with the support of the PCCs infectiologist and the Center for Tuberculosis Prevention and Control (*Centre de Lutte Anti-Tuberculeux*). As a result, 13 people were investigated, one of whom was transferred to Cayenne for further treatment.

***Managing the risk of a measles epidemic on the Brazilian border***

While several cases of measles had been reported in Oiapoque, the Brazilian town bordering Saint Georges de l'Oyapock on the French side, a sick Brazilian family visited residents of Saint Georges. The MPHT quickly organized a mission to the affected district of Saint Georges. This area was only accessible by canoe. The MPHT on-site team explained to the 19 contacts of the index case what to do in the event of symptoms. They also checked their measles vaccination status and offered an update to the nine people who were not up to date with their measles vaccination. This rapid response limited the number of secondary cases, as only one case - a cousin of the family concerned - developed non-severe measles (PCR-positive) following the intervention.

***Cases of dengue fever***

At the beginning of 2021, the dengue epidemic ended in French Guiana. In response to the diagnosis of cluster cases in the villages surrounding Grand Santi, the MPHT on-site team carried out a mission in February to raise awareness, identify and refer suspected cases to the PCC. By travelling around the villages, the team raised awareness among 11 people and did not identify any potential dengue patients. The epidemiological signal was then extinguished.

***Respiratory Syncytial Virus epidemic***

In early January, the French Emergency Medical Assistance Service (*Service d’Aide Médicale d’Urgence*) alerted the PCCs coordination to the increase in severe cases of acute respiratory distress syndrome among children in Maripa Soula. These children had to be transported urgently to Cayenne by helicopter, overflowing the evacuation system. An epidemic of Respiratory Syncytial Virus (RSV) was quickly diagnosed. Material and human reinforcements were sent to the Maripa Soula PCC. Signals indicated that remote and precarious villages were affected. In response, an assessment mission was organized in these areas. The MPHT on-site team, accompanied by a physician from the PCC of Maripa Soula, traveled by canoe to the remote villages of the Upper Maroni to assess the epidemic dynamics. With the support of village chiefs, they examined and referred 15 children to the PCC. They also advised villagers on preventive measures to follow. A second identical mission was organized five days later. This mission enabled the transfer to the PCC of Maripa Soula of 16 children with severe symptoms linked to the RSV epidemic. The door-to-door approach was essential to ensure early medical care for the children. A final mission took place on January 15 in the village of Antecum Pata. No new serious cases were reported.

**Appendix 3.**

[Insert figure]

**Figure**. Map of ethnic groups and languages in French Guiana. Realization: S. Rabier, from "Geographical distribution of ethnic groups and languages in French Guiana" (realization: S. Rabier, L. Epelboin and M.-A. Tareau) in "Overview of infectious and non-infectious diseases in French Guiana in 2022” (5)

**Appendix 4.**

[Insert figure]

**Figure.** Stages in the temporal construction of the MPHT and the training of its team members from 2019 to 2022]
